# Collective Spin-Wave Dynamics in Gyroid Ferromagnetic Nanostructures

**DOI:** 10.1021/acsami.4c02366

**Published:** 2024-04-22

**Authors:** Mateusz Gołębiewski, Riccardo Hertel, Massimiliano d’Aquino, Vitaliy Vasyuchka, Mathias Weiler, Philipp Pirro, Maciej Krawczyk, Shunsuke Fukami, Hideo Ohno, Justin Llandro

**Affiliations:** † Institute of Spintronics and Quantum Information, Faculty of Physics, Adam Mickiewicz University, Uniwersytetu Poznańskiego 2, 61-614 Poznań, Poland; ‡ Université de Strasbourg, CNRS, Institut de Physique et Chimie des Matériaux de Strasbourg, F-67000 Strasbourg, France; § Department of Electrical Engineering and ICT, University of Naples Federico II, 80125 Naples, Italy; ∥ Fachbereich Physik und Landesforschungszentrum OPTIMAS, Rheinland-Pfälzische Technische Universität Kaiserslautern-Landau, Erwin-Schrödinger-Straße 56, 67663 Kaiserslautern, Germany; ⊥ Research Institute of Electrical Communication (RIEC), Tohoku University, 2-1-1 Katahira, Aoba-ku, Sendai-shi, Miyagi 980-8577, Japan; # Center for Science and Innovation in Spintronics (CSIS), Tohoku University, 980-8577 Sendai, Japan; ¶ Center for Innovative Integrated Electronic Systems (CIES), Tohoku University, 468-1 Aramaki Aza Aoba, Aoba-ku, 980-0845 Sendai, Japan; ∇ WPI Advanced Institute for Materials Research, Tohoku University, 2-1-1 Katahira, Aoba-ku, 980-8577 Sendai, Japan; ○ Inamori Research Institute for Science, 600-8411 Kyoto, Japan

**Keywords:** gyroids, 3D nanostructures, magnonics, ferromagnetic resonance, micromagnetic simulations, spin-wave modes

## Abstract

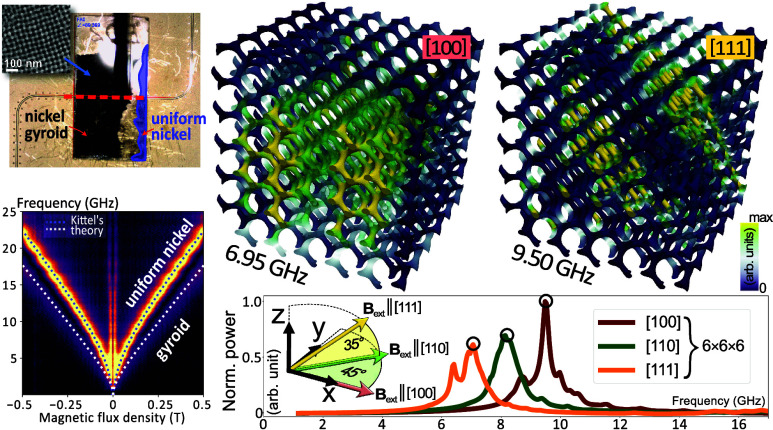

Expanding upon the burgeoning discipline of magnonics, this research elucidates the intricate dynamics of spin waves (SWs) within three-dimensional nanoenvironments. It marks a shift from traditionally used planar systems to exploration of magnetization configurations and the resulting dynamics within 3D nanostructures. This study deploys micromagnetic simulations alongside ferromagnetic resonance measurements to scrutinize magnetic gyroids, periodic chiral configurations composed of chiral triple junctions with a period in nanoscale. Our findings uncover distinctive attributes intrinsic to the gyroid network, most notably the localization of collective SW excitations and the sensitivity of the gyroid’s ferromagnetic response to the orientation of the static magnetic field, a correlation closely tied to the crystallographic alignment of the structure. Furthermore, we show that for the ferromagnetic resonance, multidomain gyroid films can be treated as a magnonic material with effective magnetization scaled by its filling factor. The implications of our research carry the potential for practical uses such as an effective, metamaterial-like substitute for ferromagnetic parts and lay the groundwork for radio frequency filters. The growing areas of 3D magnonics and spintronics present exciting opportunities to investigate and utilize gyroid nanostructures for signal processing purposes.

## 1 Introduction

Spin waves (SWs) and their intricate manipulation in magnetic materials constitute a significant part of the contemporary research. In ferromagnetic systems, the complex dynamics of SWs arises from the coexistence of magnetostatic and exchange interactions. The magnetostatic interactions, being highly anisotropic in thin structures, induce a profound dependence of SW properties on the relative orientation of magnetization and wavevector. This gives them a number of properties that are conspicuously absent in other types of waves, including negative group velocity, caustics, readily accessible nonlinearity, and dynamic reconfigurability control.^1^ The potential applications are myriad, ranging from signal propagation without Joule–Lenz heat dissipation,^2,3^ to the tunability of dispersion and group velocity.^4,5^ The construction of magnonic systems, known for their enhanced efficiency, further emphasizes the unique properties of SWs and provides a compelling rationale for their extensive exploration.^2,6,7^ Thus, the focus of current research is to harness and adapt magnetization dynamics for sophisticated industrial applications, a goal underscored by recent advances and roadmaps.^8,9^

Nanostructured 3D networks may give rise to topological and geometrical effects and emergent material properties, offering new possibilities for SW manipulation.^10−12^ A fully interconnected 3D system opens up a new degree of freedom for novel phenomena, allowing interactions and collective effects in all three dimensions.^13−15^ In recent years, there has been significant development of fabrication techniques such as two-photon lithography, focused electron beam deposition, and block copolymer templating, which now allow the fabrication and measurement of complex 3D structures on the nanometer scale.^16−19^

This paper analyzes a promising yet hardly explored structure in magnetism called a gyroid, which was discovered and first presented in 1970.^20^ It is defined by chiral triple junctions and periodicity in all three spatial directions, classified as *I*4_1_32 space group (no. 214).^21^ In recent years, many studies have been published describing gyroids in the field of photonics, where they have been presented as potential chiral beamsplitters,^22^ nonlinear optical metamaterials,^23,24^ or photonic crystals.^25−27^ It has also inspired many research groups into the fabrication of artificial systems based on this geometry.^22−24,28−34^ The 3D structural unit of magnetic gyroids is in nanoscale and has both chirality and curvature, which has been shown to be highly effective in controlling noncollinear spin textures.^35^ Recently, numerical and experimental visualization of the magnetic structure in a single and double Ni_75_Fe_25_ gyroid network has also been performed.^16^ The effective Dzyaloshinskii–Moriya interaction and the curvature-related anisotropy are further important research topics in this context.^36−39^ Curved magnetic wires and films also exhibit novel physical effects,^40^ which together with chiral and topological properties,^41,42^ open new avenues for future research.

SWs have been extensively researched in 2D structures; however, there are limited studies on artificial ferromagnetic systems in full 3D^43,44^ and a lack of experimental research on the collective dynamics of SW in 3D nanostructures. Gyroids, due to their unique geometry and dimensions comparable to the exchange length, bear multidimensional properties of interactions with SWs through shape anisotropy, an inhomogeneous demagnetization field, and chirality. They are an intriguing candidate for the realization of artificial 3D magnonic crystals with highly coupled geometric and magnetic states.^12,45−47^ Furthermore, gyroids, with a significant number of energetically equivalent stable states, may have the potential for artificial spin ice systems and active elements in unconventional computing architectures.^48^

Approaching these structures from a magnonics perspective, we employ micromagnetic simulations to delve into the collective SW dynamics. Our investigation scrutinizes the alignment of the external magnetic field in relation to the gyroid’s crystallographic axes, offering an insightful perspective on the impact of their geometry on shaping collective magnetization dynamics. To complement our simulation-based findings, we performed experimental broadband ferromagnetic resonance (BBFMR) measurements on the nickel (Ni) gyroid structure. This empirical approach enhances our numerical observations, specifically in terms of the frequencies and intensities of resonances excited at a given external magnetic field and the impact of various crystallographic domains on the half-width of the signals measured in the sample. Furthermore, the measured magnetic field dependencies suggest that the gyroid structure exhibits magnonic metamaterial-like properties. This combination of simulation and empirical experimentation fosters a comprehensive understanding of the multifaceted dynamics within these unique 3D nanostructures, highlighting their potential applications and motivating further research.

## 2 Geometry and Material Parameters

The gyroidal surface was given for the first time using conjugate surface construction,^49^ and in ref (50) its embedding was subsequently proved. The volume fractions of minimal and constant mean curvature gyroids have been further investigated numerically^51^ with the constant mean curvature variants of the geometry.

**Figure 1:**
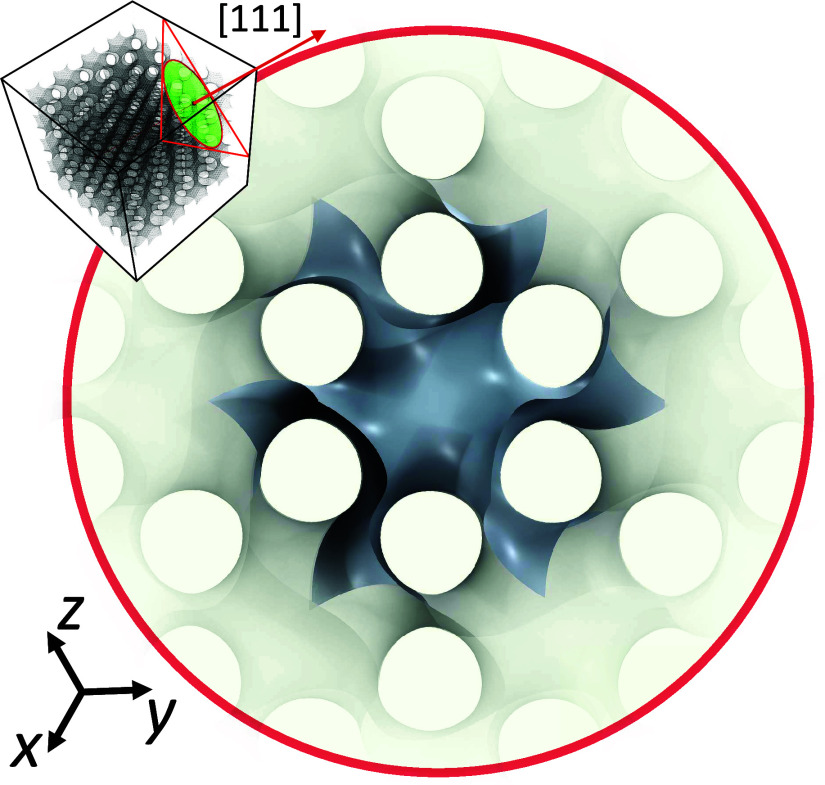
Representation of the gyroid surface model (ϕ = 0%), highlighting the unit cell structure. The illustration accentuates the unit cell configuration along the crystallographic [111] direction in orthographic projection, unveiling the hexagonal organization inherent in the gyroid’s channels.

In other fields, the gyroid is referred to as Laves’ graph of girth ten^52^ and the *K*_4_ crystal.^53^ It consists of cubic unit cells composed of triple bonds connected by nanorods with elliptical cross sections for the nonzero volume filling fraction ϕ, the range of which is described in ref (24). For ϕ = 0% (see Figure 1), a gyroid surface divides space into two labyrinths of paths oriented in opposite directions. The empty, unobstructed channels pass through the gyroid labyrinths in directions [100] and [111], and the paths emerge at 70.5° angles to any given channel as it passes through. Circling or gyrating down the channel in this way gives rise to the term “gyroid”. Interestingly, gyroids exist in several Schwarz surface families that preserve different symmetries of triply periodic minimal surfaces and, like many others, can be approximated by a trigonometrical equation
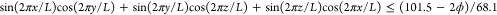
1where *L* is the gyroid’s unit cell length.

The studied gyroid nanostructure was constructed by solvent vapor annealing, selective dissolution, and electrodeposition of a block copolymer template, following the protocol described in ref (16). The gyroidal structure was fabricated by applying polyisoprene-*b*-polystyrene-*b*-poly(ethylene oxide) (ISO) triblock terpolymers with block volume fractions of ϕ_PI_ = 0.30, ϕ_PS_ = 0.53, and ϕ_PEO_ = 0.17 to fluorine-doped tin oxide (FTO)-coated glass substrates. Initial cleaning with Piranha solution at 80 °C for 15 min prepared the substrates, which were then treated with octyltrichlorosilane. A 1 μm BCP film was spin-coated from a 10 wt % anhydrous anisole solution and formed into the gyroid morphology by solvent vapor annealing under nitrogen saturated with chloroform vapor at 26 °C. After annealing, the PI block was removed by UV exposure and immersion in ethanol to form gyroid polymer templates. Ni electrodeposition from a commercial solution followed in a three-electrode cell, filling the voided single-gyroid network with Ni under a constant potential at −1.05 V while monitoring the deposition charge. This method efficiently produces gyroid structures with precise Ni insertion. The material parameters used in the micromagnetic simulations represent those of Ni utilized in the fabrication of the sample, i.e., saturation magnetization *M*_s_ = 480 kA/m, exchange stiffness *A*_ex_ = 8.6 pJ/m, and *g*-factor equal to 2.14.^54^ In each relaxation simulation, the Gilbert damping was set to a large value of α = 0.5 to obtain fast convergence to the equilibrium state. Then, for all frequency-domain simulations, this parameter was set to a low value of 0.01.

**Figure 2:**
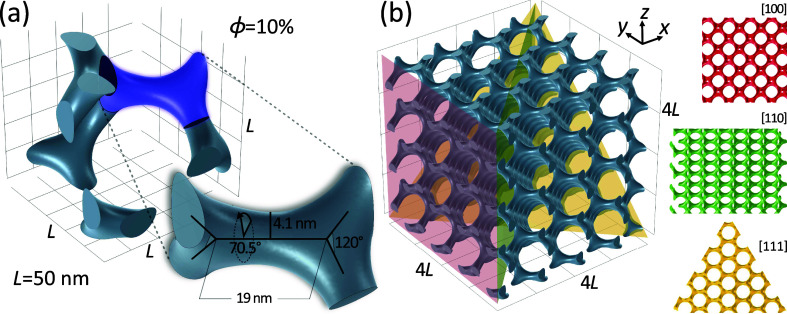
Depiction of the gyroid unit cell and geometric modeling for micromagnetic simulations. The focus of our investigation is a cubic gyroid unit cell measuring *L* = 50 nm, featuring a volume fraction ϕ = 10% as depicted in (a). For the purpose of micromagnetic simulations, a geometric model consisting of an aggregate 4 × 4 × 4 unit cells, or equivalently 200 × 200 × 200 [nm], was employed, as displayed in (b). Illustrated alongside are the three principal high-symmetry directions of the gyroid structure ([111]—yellow triangle, [110]—green rectangle, and [100]—red square) which are color-coordinated to match the planes intersecting the structure. Each direction reveals the unique distribution and shape of the gyroid’s channels: the [111] direction displays a hexagonal pattern and round holes, the [110] direction exhibits a square pattern and lenticular holes, while the [100] direction showcases a square pattern and round holes.

The unit cell of the investigated gyroid sample measures 50 nm in each direction and has a volume fraction (ϕ) of approximately 10%, which here corresponds to a cross-sectional radius of a single gyroid node of about 4.1 nm (where it is oval in shape) and an arm length of about 19 nm. The geometric parameters of the gyroid unit cell are described in Figure 2a and are the same for all models analyzed in this work. In the experimental portion of this investigation, the gyroid sample comprises 12 unit cells of height, resulting in an overall thickness of 600 nm. Consequently, individual strut diameters are on the order of single nanometers, mirroring intrinsic magnetic length scales, including exchange length, domain wall width, and SW wavelength.

## 3 Micromagnetic Simulations

To unravel the magnetic phenomena transpiring within nanoscale gyroidal struts, we perform comprehensive micromagnetic simulations of the system, taking into account dipole and exchange interactions. In this endeavor, we harness the capabilities of our GPU-accelerated open-source finite-element (FEM) micromagnetic solver *tetmag*.^55^ A remarkable feature of *tetmag* is its proficiency at resolving magnetostatic open boundary problems in large-scale micromagnetic simulations via a hybrid finite-element/boundary-element (FEM-BEM) formalism.^56^ Notably, we forego the assumption of periodic boundary conditions in all of the simulations presented herein.

**Figure 3:**
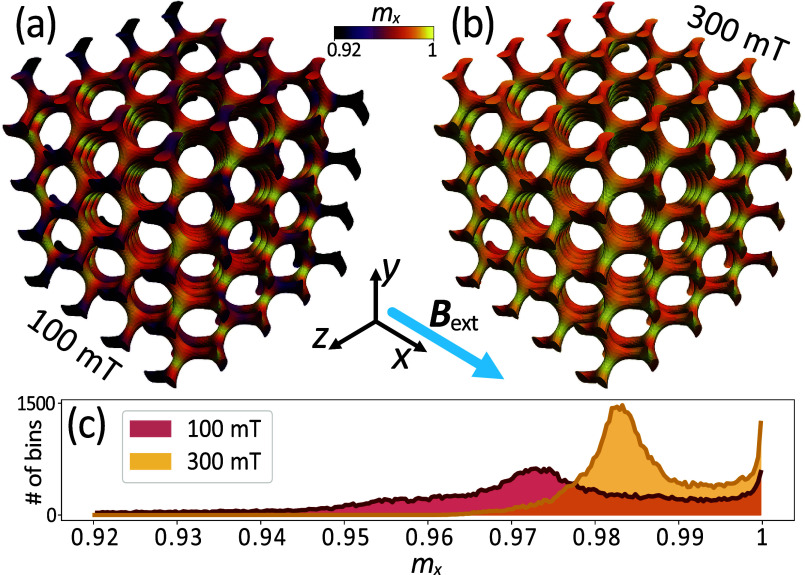
Images of the postrelaxation static configurations of the reduced magnetization component *m*_*x*_(≡*M*_*x*_/*M*_s_) parallel to the external magnetic field, aligned in the crystallographic direction [100]. Panel (a) displays the case when *B*_ext_ = 100 mT. The deviation of the magnetization vector from the *x*-direction is notably larger compared to (b) with *B*_ext_ = 300 mT, where the magnetization distribution is nearly uniformly oriented along the direction of the external magnetic field. (c) Histogram (number of numerically calculated magnetic moments falling within specific ranges of *m*_*x*_) of the reduced magnetization component distribution *m*_*x*_ in the simulated gyroid model for both magnetic field magnitudes. The number of bins indicates the number of elementary simulation elements (tetrahedrons).

All of the following micromagnetic simulations are performed in two steps, where the first step is to calculate the stable (relaxed) magnetic configuration at a given field strength (see, e.g., Figure 3a,b). After the magnetization relaxation, we performed simulations of the ferromagnetic resonance using a dedicated frequency domain algorithm^57^ based on a formulation proposed by d’Aquino et al. in ref (58).

The low-amplitude alternating, and homogeneous in space, magnetic field applied in the frequency domain simulations generates a response of the magnetic system in the form of stationary magnetization oscillations with the frequency of the applied field. The magnetic susceptibility χ(ω) describes the frequency-dependent relation between the externally applied oscillating field δ**H** and the dynamic component of magnetization δ**M**. For a more detailed discussion of the dynamic susceptibility and its definition, see ref (57).

**Figure 4:**
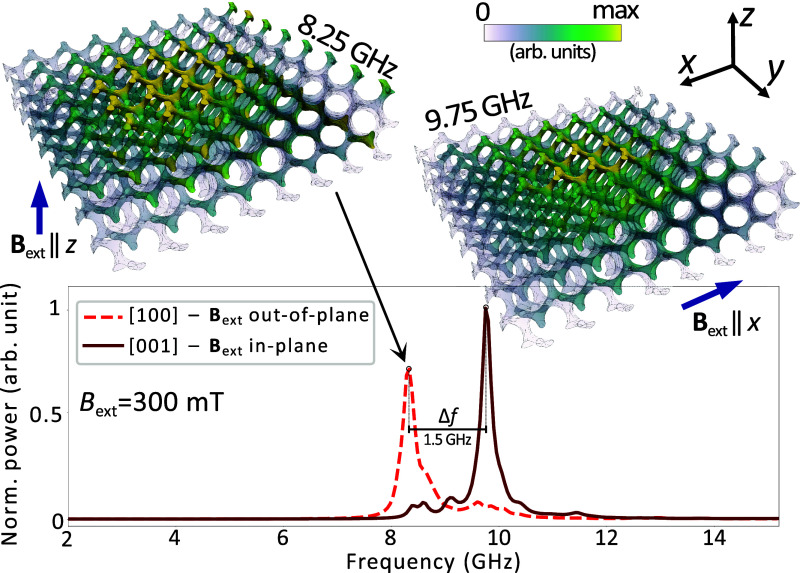
Resonance frequency spectra of 8 × 8 × 2 gyroid structures obtained from micromagnetic simulation. The graphical representations showcase the resonance derived from two different orientations of an applied magnetic field: a dashed line represents the signal from a sample subjected to a 300 mT field, directed out-of-plane (along the *z*-axis); the solid line, meanwhile, illustrates the response of the sample magnetized in the in-plane (*x*-axis) direction. The employed color scale is representative of the imaginary part of magnetic susceptibility.

**Figure 5:**
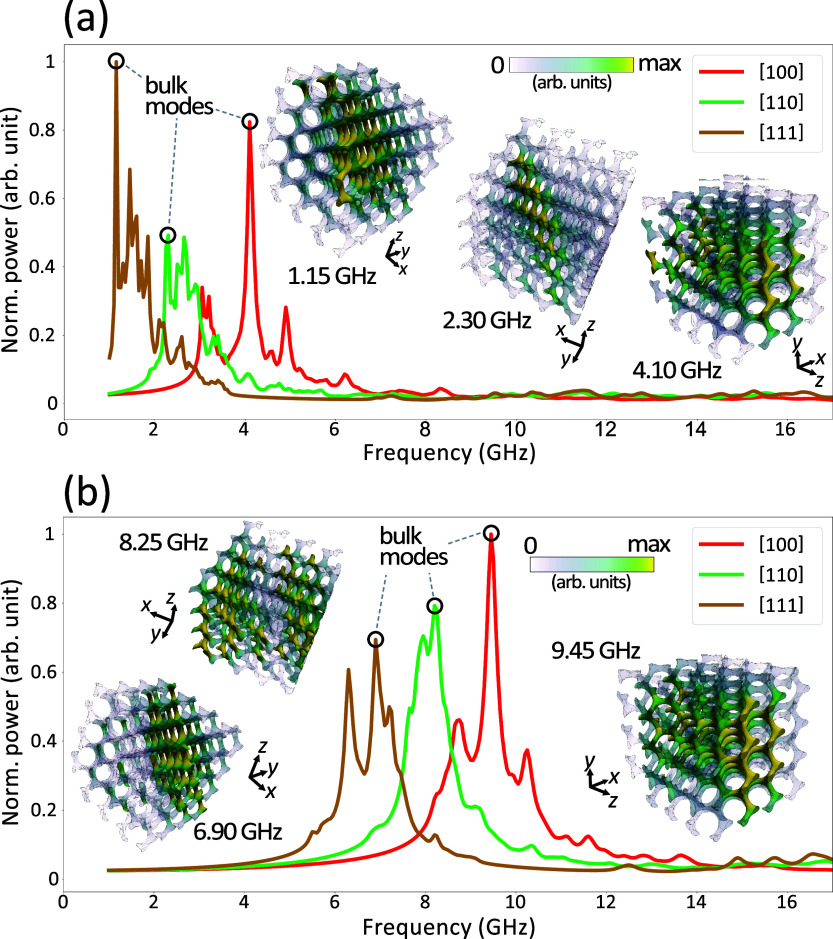
Micromagnetic simulation-derived resonance frequency spectra for 4 × 4 × 4 gyroid constructs. The spectra are derived from two scenarios: (a) in which the applied external magnetic field has a strength of 100 mT and (b) where it measures 300 mT. Within each plot, different color coding indicates the crystallographic direction in which the field is applied, with the specific points encircled on the graph signifying the ferromagnetic resonance. Visual illustrations and resonance frequency values, in sequential order of their appearance, are exhibited as insets within the plots. The coloring scheme used here corresponds directly to the imaginary component of the magnetic susceptibility.

**Figure 6:**
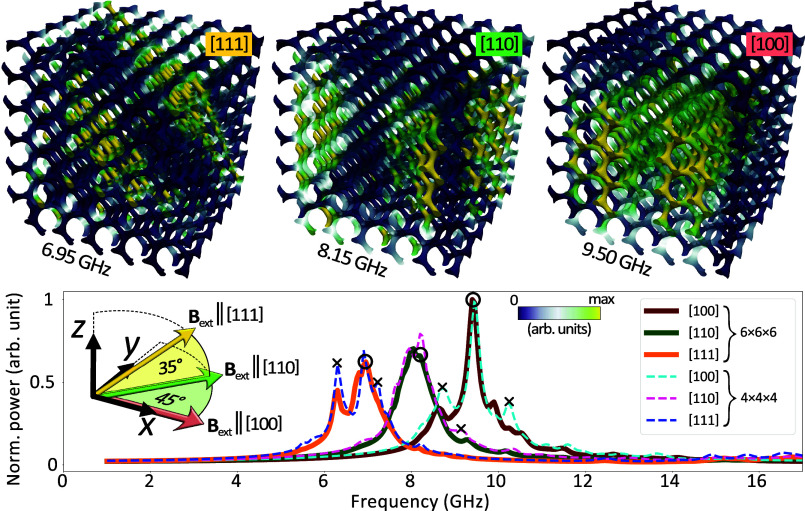
Spectral examination of high-intensity volume modes within a 6 × 6 × 6 gyroid structure. The lower section of the figure presents a plotted distribution of the frequency spectra, with the high-intensity volume modes depicted in the upper part distinctly marked by black circles. For the purpose of comparison, spectra corresponding to more compact structures (illustrated as dotted lines) are superimposed on the graph, thereby clearly demonstrating a marked decline in the intensity of edge modes (denoted with crosses) commensurate with the enlargement of the structure dimensions. The color gradation utilized in the visual representation of the modes is proportional to the imaginary component of the magnetic susceptibility.

In the linear-response theory, the imaginary component of the susceptibility is related to dissipative processes in which the sample absorbs energy from the applied field. These absorption peaks denote resonances and generally coincide with frequencies of the maximum oscillation amplitude of the dynamic magnetization. For the case of a spatially homogeneous harmonic magnetic field applied in the simulations, we can thus analyze the spatial distribution of the magnetization oscillation amplitude at these absorption peaks to identify the modes developing at these resonances. The colors in the mode visualizations (Figures 4–6) refer to the imaginary magnetic susceptibility component of the gyroid structure, which in this case is analogous to the modulus of the dynamic components of the magnetization.

**Figure 7:**
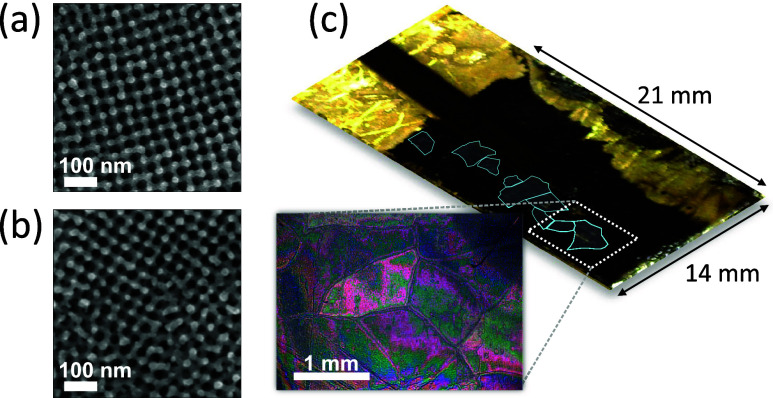
Multidomain gyroid structure employed in BBFMR measurements, illustrating the complex and varied nature of the sample. In panels (a) and (b), scanning electron microscope (SEM) topographical images offer detailed views of two distinct sample regions, characterized by different crystallographic directions and varying degrees of amorphousness. Panel (c) showcases a photograph of the entire sample, annotated with the approximate locations of several prominent domains. These domains were identified and characterized through polarized light microscopy.

Initially, our simulation work involves the comparative analysis of the ferromagnetic response within the planar system geometry for both in-plane and out-of-plane directed fields to ascertain if a planar gyroid network exhibits macroscopic shape anisotropy. Our experimental sample, possessing a near-planar macroscopic geometry, boasts a lateral dimension spanning a few millimeters and a thickness in the submicron range (see Figure 7).

Therefore, first we analyze a quasi-planar 8 × 8 × 2 gyroid model (number of the unit cells along the *x*, *y*, and *z*, respectively) to verify the influence of external shape anisotropy on resonant frequencies. The model here is 400 × 400 × 100 nm in size (see Figure 4) and consists of about 366,000 tetrahedral discretization cells (∼2860 per unit cell). We consider two cases where the external magnetic field is directed out-of-plane and in-plane: [100] and [001], respectively, which are crystallographically identical. We see in Figure 4 an apparent anisotropy in the spectrum that changes in both field directions. The maximum absorption frequency shifts from about 8.25 GHz in the out-of-plane scenario to about 9.75 GHz in the in-plane scenario (Δ*f* = 1.5 GHz). It can therefore be concluded that when simulating gyroids in a cubic simulation volume, as shown in Figure 2b, we can expect resonant frequencies with values lower than those obtained from experiments due to the significant influence of macroscopic shape anisotropy. The most accurate results of micromagnetic simulations could be obtained by simulating a much larger structure. However, due to limited computational resources and simulation time, it was necessary to limit the size of simulated structures.

To study gyroid-based effects at the nanometer scale, the model with dimensions of 4 × 4 × 4 unit cells (200 × 200 × 200 nm; see Figure 2b) was used in the second stage of micromagnetic simulations, consisting of about 171,000 tetrahedral discretization cells (∼2670 per unit cell). The cubic shape of the structure allows a more accurate analysis of the dynamic magnetization distribution and insights into the effects associated with gyroid crystallography.

In this examination, we scrutinize three crystallographic directions, namely, [100], [110], and [111], as illustrated in Figure 2b. We further engage with two distinct magnitudes of the external static magnetic field *B*_ext_, set at 100 and 300 mT, as depicted in Figure 5. The considerable computational power and time demanded by the simulations of such complex structures necessitate this restricted selection of parameters.

As shown in Figure 5, magnetic resonance simulations of gyroid structures reveal their pronounced geometric anisotropy. Upon juxtaposition of these plots with those for the planar structure depicted in Figure 4, Figure 5 exhibits a substantially richer spectrum for both external magnetic field values. This richness originates from the increased prominence of edge modes within the overall signal, a characteristic feature of cubic structures. Visual representations of these can be accessed in the Supporting Information (Figures S2 and S3). Through the identification and visualization of bulk modes, we observe that these predominantly emerge within the structure’s inner part. This observation suggests that their existence can be primarily attributed to the intrinsic gyroid geometry rather than edge features or artifacts produced in the cutting region, underscoring the distinctive and influential role that the gyroid geometry plays in modulating the magnetic response of these 3D structures. Consequently, our analysis focuses solely on the resonant modes linked to the volumetric portion of the structure, disregarding the discernible satellite peaks attributable to the edge modes.

The resonance spectra findings unequivocally assign the shifting resonance signals observed in the rotating gyroid sample to the crystallographic anisotropy, as evidenced by the plots displayed in Figure 5. A comparative assessment of the plots corresponding to 100 mT in Figure 5a and 300 mT in Figure 5b, reveals a discernible increase of frequencies. However, the order and dispersion of the resonance frequency peaks remain invariant, with the two most distal bulk modes (1.15 GHz, 4.10 GHz in Figure 5a and 6.90 GHz, 9.45 GHz in Figure 5b) separated by 2.95 and 2.55 GHz, respectively. Those separations emphasize the pivotal role played by gyroid geometry in dictating the system’s magnonic properties, demonstrating that these features are not merely a consequence of extrinsic variables.

In the final stage of our simulations, we conducted an analysis of a larger cubic gyroid structure. In the course of visualizing the modes via micromagnetic simulations (Figure 5), we uncovered a compelling localization of high-amplitude magnetization precession. In order to enhance the visibility of these modes vis-à-vis smaller structures and to incrementally amplify the signal of volume modes in relation to satellite ones, we proceeded to simulate and scrutinize a gyroid structure comprising 6 × 6 × 6 unit cells. This extensive structure was discretized into upward of 638,000 finite elements (∼2950 per unit cell). The resultant magnetization intensity distribution (Figure 6 top row) distinctly delineates planes orthogonal to the 300 mT external magnetic field axis, manifest at modes 6.95 GHz for [100], 8.15 GHz for [110], and 9.50 GHz for [111]. Furthermore, in the case of the field-aligned along [100] and [110], the modes manifest a periodicity (see the top row showing the spatial distribution of the imaginary component of the magnetic susceptibility), a characteristic not previously discernible in the 4 × 4 × 4 structure. The graph depicted at the bottom of Figure 6 showcases the FMR spectrum derived from simulations of the 6 × 6 × 6 model across the three investigated crystallographic directions. To facilitate comparison with corresponding calculations for a more diminutive structure (Figure 5b), the outcomes of these simulations were superimposed on the graph as dashed lines. It becomes evident that the influence of edge modes (denoted with a cross) in most cases diminishes in correlation with the model dimensions’ expansion, yielding a decrease of about 28% when increasing the layout from 4 × 4 × 4 to 6 × 6 × 6 unit cells, i.e., 3.375-fold. The normalized intensity of the volume modes (denoted by a circle) remains unchanged for the cases [100] and [111], but is slightly reduced for [110], most likely due to its coupling with the edge mode and/or its localization at the corners of the structure.

## 4 Experiment

The main conclusion elucidated from our simulations is the intrinsic relationship between the ferromagnetic response of the gyroid network and its orientation to the axis of the static magnetic field. Figure 7 indicates that the sample embodies multiple domains, manifested as gyroid patches with varying crystallographic orientations relative to a consistent reference point. The varied orientations significantly influence the material’s magnetic response, adding complexity to its behavior in a magnetic field.

In Figure 7b, a distinct transition is observed from top to bottom, ranging from a well-defined, highly oriented region to an irregular, less-structured domain. This transition can be construed as the domain wall within the complex gyroid structure. As shown in the simulations, each distinct crystallographic orientation of the gyroid with respect to the externally applied magnetic field may lead to a nuanced alteration in the resonance response. Owing to the substantial multiplicity of domains, the anticipated FMR signal will manifest as an averaged and diluted amalgamation of contributions from individual domains.

Through the application of polarized light spectroscopy, the research facilitated the estimation of the individual domains of the gyroid structure; that is, the regions exhibiting homogeneous crystallographic alignment. As depicted in Figure 7c, the dimensions of the largest domains extend to the millimeter scale. However, it must be acknowledged that these particular measurements do not allow for the determination of the specific crystallography present within each domain. In an effort to achieve a dynamic magnetic characterization of the Ni gyroid sample, BBFMR measurements were executed in the frequency range of 0.1 to 25 GHz, following the methodology delineated by Heinrich.^59^ This involved the utilization of a two-port vector network analyzer, with connections established to opposite ends of a coplanar waveguide (CPW) in accordance with the techniques described by Montoya.^60^

**Figure 8:**
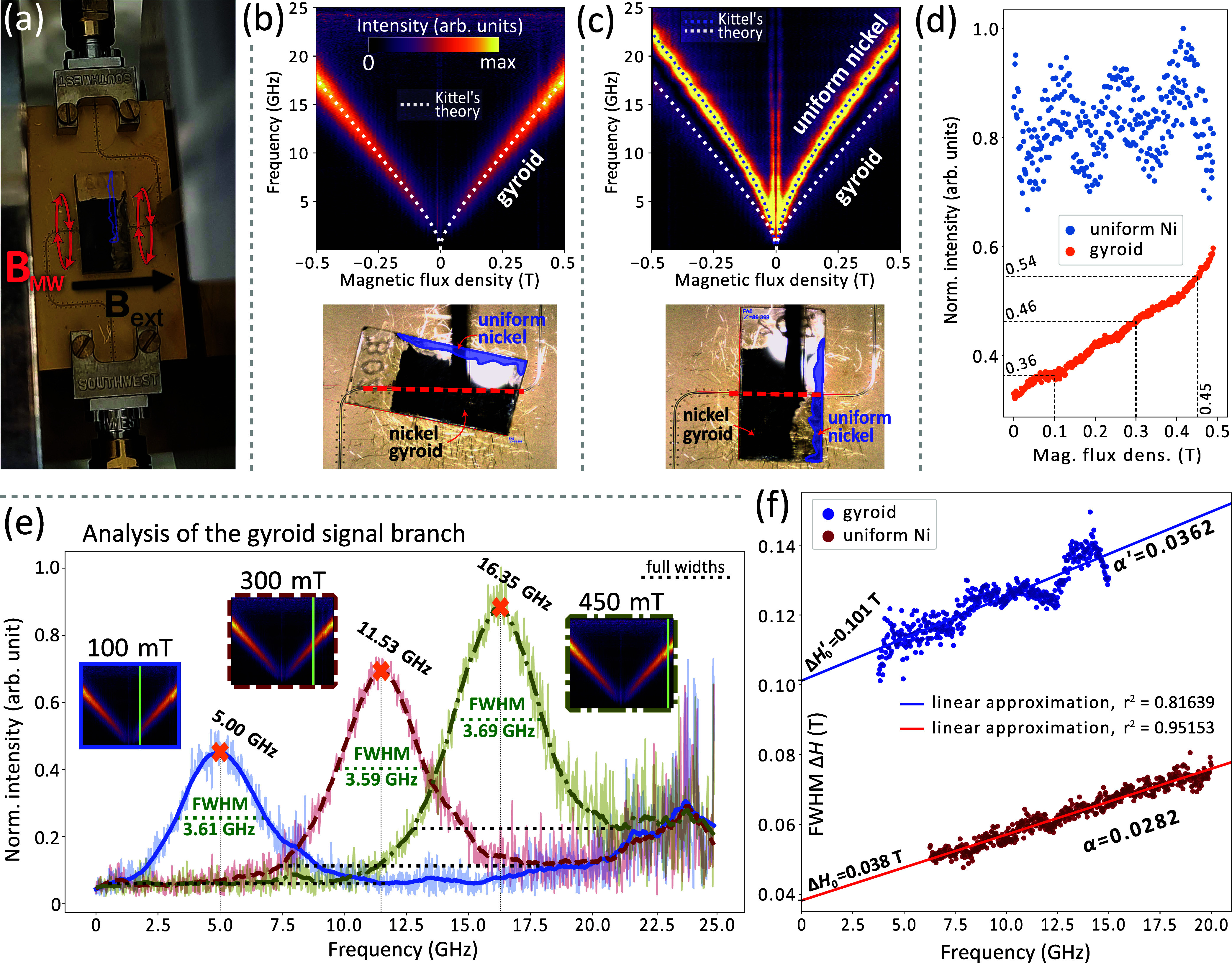
BBFMR measurement conducted on the Ni gyroid structure. The sample underwent repositioning with respect to the CPW to elucidate the effect of an additional homogeneous Ni layer present within the specimen. In two distinct configurations, separate assessments were made of the energy absorption stemming from the microwave field *B*_MW_, applied perpendicular to the external static magnetic field (a). The resultant plots of dynamic magnetization amplitude as functions of static magnetic flux density and frequency for selected sample configurations are depicted in (b,c). These render a conspicuous signal attributed to the gyroid layer when the CPW is in direct alignment below it (b), and an additional, higher-frequency signal emanating from the uniform Ni layer (c) when the CPW (as delineated by the red dashed line) intersects its projected position (highlighted in purple on the sample). The dotted lines in the graphs represents the theoretical fit derived from the Kittel formula (see eq 2). For uniform Ni, parameters from micromagnetic simulations were used, while for gyroid, we implemented the calculated effective parameters, i.e., saturation magnetization *M*_eff_ = 132 kA/m, and the *g*-factor of 2.2. Plot (d) shows a summary of the peak intensities calculations of FMR signals (blue dots for uniform Ni, and orange dots for gyroid) as a function of the external magnetic field strength. Normalized intensity values for 100, 300, and 450 mT fields are indicated. In graph (e), a cross-sectional analysis of the BBFMR signals is depicted for distinct values of the external magnetic field, where the solid blue line corresponds to *B*_ext_ = 100 mT, the dashed brown line to *B*_ext_ = 300 mT, and the dash-dotted green line to *B*_ext_ = 450 mT. Additionally, horizontal dashed green lines mark the full width at half-maximum (FWHM) for each section, providing quantitative insights into the resonance line widths along with their respective values. The orange crosses signify peak maxima and their corresponding frequencies, pinpointing the resonant behavior within the explored frequency range. Insets furnish intensity plots from the BBFMR measurements, with the green vertical lines highlighting the specific locations of the sections for each magnetic field value. Finally, plot (f) shows the magnetic field FWHM’s as a function of frequency for BBFMR signals of gyroid (purple dots) and uniform Ni (dark red dots). Based on the experimental data and using eq 3, a linear regression was performed and the values of the determination coefficient *r*^2^, Δ*H*_0_ (from the abscissa of the lines) and the eq 3-derived damping values α (from the slope of the lines) were estimated. Parameters related to the gyroid structure are marked with a prim (′).

The gyroid probe was mounted on a CPW with a 80 μm wide center conductor facing downward to ensure maximum coupling with the microwave field^61^ and the static external magnetic field applied within the sample plane, along the CPW center conductor (Figure 8a). We tested several orientations of the specimen with respect to the CPW line and discovered a signal from both gyroid-structured Ni and a uniform layer of this material (Figure 8b,c). Measurement data from more sample orientations relative to CPW are provided in the Supporting Information (Figure S1). Indeed, the precise manipulation of the sample’s position on the CPW elucidated the presence of a higher frequency signal that is uniquely associated with the homogeneous Ni layer present at one of the sample’s edge. This distinct relationship was substantiated by an excellent agreement between the observed signal and the theoretical prediction derived from the Kittel formula
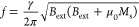
2where *f* is the resonance frequency, μ_0_ is a vacuum permeability (∼4π × 10^–7^ T m/A), and γ is the gyromagnetic ratio. For the comparative calculations, we employed identical magnetic parameters as in the numerical simulations, specifically, the *g*-factor of 2.14 and saturation magnetization *M*_s_ = 480 kA/m. Contrarily, the lower frequency signal is consistently identified with the gyroid-structured region of the sample, a correlation supported by its sustained presence irrespective of orientation relative to the CPW.

Using eq 2, we not only fitted the resonance signal derived from the homogeneous Ni structure but also attempted to fit it to the experimental signal in the gyroids and estimated the effective values of the saturation magnetization and the *g*-factor.

As a result, a satisfactory fit of the Kittel curve to the gyroidal resonance signal for different sample orientations relative to the CPW (see Figure S4 in the Supporting Information) was obtained using *M*_eff_ = 0.275*M*_s_ = 132 kA/m, and *g*_eff_ = 1.028*g* = 2.2, as shown in Figure 8b,c with a white dotted line. Such findings suggest that the given 3D structure exhibits magnonics’ metamaterial-like properties, so far considered only for the planar structures,^62−66^ capable of modulating (specifically, decreasing) the effective saturation magnetization and the dynamical response of the structure in a methodical and foreseeable manner. The effective magnetization, which is higher than expected with only a 10% filling fraction, implies that the gyroid structure may have anisotropy. It can be indicative of a strong shape anisotropy inherent to the particular nanoelements of this structure, which ‘anchors’ the magnetization in place until it is forced to switch due to a change in the field direction.

To facilitate a qualitative comparison between the results of the experiment and simulation, we examined the FWHM’s. Figure 8 reveals that the signal from gyroid exhibits a broader frequency FWHM than in Ni, e.g., at 450 mT field it is 3.69 GHz for gyroid and 3.09 GHz for homogeneous Ni. This phenomenon is likely attributable to the multidomain nature of the sample. That is, the signal observed from the gyroid is the average value coming from several different domains located above the CPW and differing in crystallographic orientation. We deduce from the BBFMR measurements depicted in Figure 8e an average difference between the FWHM’s of 1.3 GHz over different values of the external magnetic field. The micromagnetic simulations for the cube shape (Figure 5) reveal a maximum peak separation of 2.55 and 2.95 GHz for the field of 300 and 100 mT, respectively (in both cases between [100] and [111] crystallographic directions). Our simulations meticulously evaluated the crystallographic directions that represent the most disparate configurations of the gyroid lattice relative to the field, most likely resulting in the largest feasible separation of resonant frequencies.

In field-swept FMR experiments conducted at a fixed frequency, the absorption line FWHM conforms to μ_0_Δ*H* = 4πα*f*/γ. This occurs when the magnetization vector is aligned with the applied magnetic field, either in the plane or perpendicular to it. Such alignment results in a line width that scales proportionally with frequency, where the slope of this scaling is defined by the Gilbert damping parameter α.^67^ Beyond this intrinsic contribution, empirical data also indicate the presence of a frequency-independent term
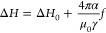
3denoted by Δ*H*_0_ that represents the inhomogeneous contributions, which add to the overall line width observed in the experiments.

Experimental investigations have enabled a linear regression analysis of FWHM across a spectrum of *B*_MW_ frequencies, as depicted in Figure 8f for the designated orientations of the sample over CPW. This analytical approach is instrumental in ascertaining the damping values α and the inhomogeneous line width contribution Δ*H*_0_ for both the homogeneous Ni layer and the gyroidal structure, respectively, employing eq 3. In the first case, the derived values are α = 0.0282 ± 2.54% and Δ*H*_0_ = 0.038 ± 1.28% T, with a coefficient of determination of *r*^2^ = 0.95153. Comparatively, the gyroidal structure exhibits α′ = 0.0362 ± 6.49% and Δ*H*_0_^′^ = 0.101 ± 1.05% T, with *r*^2^ = 0.81639. The damping constants for Ni align with previously reported values.^68^ The 95% confidence interval was used to calculate the range of estimates for the values. Larger α′ for the gyroid structure is likely attributable to the scattering of SW modes within the nanowires, which are much thinner than the bulk Ni and exhibit complex noncollinear interconnections. This difference varies with the sample’s orientation relative to the CPW, as further exemplified in the Supporting Information (Figure S4). The examples there, however, are subject to considerable uncertainty due to the nonlinearity of Δ*H*(*f*) for gyroid structures.

The disparity in the Δ*H*_0_ values between the homogeneous Ni layer and the gyroidal structure is also noteworthy. Such variations in the material’s magnetic properties, including anisotropy, manifest as a frequency-independent line width.^67^ The pronounced Δ*H*_0_^′^ in gyroid structures corroborates the influence of crystallographic orientation on the system’s resonant frequencies, as confirmed by micromagnetic simulations and indicated in the earlier discussion. It is that, experimentally, in a multimode sample scenario depicted in Figure 7, the inhomogeneous contribution to the effective FWHM is an aggregate effect of the various crystallographic orientations present, and Δ*H*_0_^′^ emerges from the superposition of all resonant peaks within the spectrum bounded by the extremities observed in Figures 5 and 6 (the maximum separation between FMR peaks for the studied crystallographies in the 100 mT field is 2.95 GHz, while in the 300 mT field, 2.60 GHz).

The next step is to compare the frequencies. In the experiment, the maximum intensity at *B*_ext_ = 300 mT is at 11.53 GHz (Figure 8d), while in the simulations for the gyroid of cubic shape, it is at 8.25 GHz for the [110] field orientation (among the orientations analyzed, in accordance with the above discussion about FWHM, we chose the orientation with the medium frequency, Figure 5b), giving a difference of 3.28 GHz. At 100 mT this difference decreases to 2.7 GHz (Figure 5a). The results in Figure 4 indicate that the macroscopic shape of the gyroid contributes to the results. In these simulations, considering a relatively small thin cuboid shape, the FMR frequency separation between orthogonal field orientations is Δ*f* = 1.5 GHz. However, when considering a bulk thin film ferromagnet of *M*_eff_ = 132 kA/m, the maximum influence of shape (i.e., FMR between in-plane and out-of-plane magnetic field orientations) can reach over 4 GHz. Therefore, it is reasonable to expect that the resonant frequencies in simulations will be lower than those obtained in experiments due to the significant influence of the macroscopic shape anisotropy.

A noteworthy distinction between the gyroid and homogeneous Ni signals also arises with respect to their measured intensities as a function of the external magnetic field. In the case of the gyroid structure, it shows an almost linear increase (Figure 8d), giving an almost 30% increase in intensity between 100 and 300 mT magnetic fields (and 50% between 100 and 450 mT), while for homogeneous Ni it remains fairly irregular. This intriguing behavior may reflect the intricate influence of the complex gyroid geometry on the internal demagnetization field and magnetization orientation. Such complexity may counteract the orthogonal orientation of the dynamic magnetization components with respect to the static field, thus attenuating their detectability by the microwave field, *B*_MW_. Upon increasing the field, the sample leads to a parallel orientation of the magnetization vectors, as shown in Figure 3c, thereby increasing the detectability of the magnetization precession at resonance. From this graph it can be seen that most of the bins (elementary simulation cells) are almost completely saturated for a field of 100 mT (for example, at least 94% saturation has been reached in 89% of the gyroid volume: 56,463 bins, and at 300 mT the >94% saturation is already in 100% of the structure: 63,274 bins). However, a comprehensive understanding of the FMR intensities in gyroids for larger fields would require further investigation that falls outside the scope of this paper.

## 5 Discussion

Owing to the inherently multidomain architecture of the sample under investigation, the task of identifying a singular dominant crystallography and quantifying its direct influence on the resonance spectrum presents a formidable challenge. Recognizing this complexity, the study has embraced a numerical approach, rigorously testing the three selected field directions ([100], [110], and [111]) in the finite-size gyroid, to elucidate the extent to which the structure’s intrinsic complexity governs resonance frequency variations. It is imperative to note that beyond the irregular shape, size, and interfaces of the domains, which relate to the shape anisotropy, fabrication of such an intricate structure encompasses numerous factors that elude accurate prediction and, consequently, integration within the simulation framework. Such factors include, but are not limited to, irregularities and impurities in both the shape and thickness of the nanorods, compounded by a paucity of definitive information concerning the sample’s dominant crystallographic orientation in each experimental configuration. A full experimental validation of the phenomena predicted by our micromagnetic simulations is beyond the scope of this work. However, we expect that these effects will soon be observable in experiments with directed self-assembly gyroids.^69^ Our experimental results provide the basis for this since the signs of the predicted phenomena have already been detected.

The research presented here highlights several emerging avenues for the application and practical use of 3D gyroidal nanostructures. By controlling ϕ, one could modulate the effective saturation magnetization, offering magnonic metamaterial of effective dynamical properties. Traditionally, the construction of artificial photonic or phononic crystals requires the use of different materials, each characterized by unique properties, e.g., dielectric constant or elastic properties. However, our results suggest that a gyroidal structure with a tunable filling factor could serve as an effective substitute for 3D magnonic crystals. This paradigm shift in fabrication methodology heralds a transition from 2D to 3D magnonic nanostructures with a wide range of novel applications and functionalities.

Due to the random distribution, shape, and size of the gyroid domains in the experiment, the sample can be approximated to a porous structure when examined collectively.^70^ This simplification, however, nullifies the interpretation of the relationship between the field direction and the frequency of SWs. This may ultimately be shown in future studies e.g. by using Brillouin light scattering for individual gyroid domains.

Some of the visible SW bulk modes presented in this work show also an intriguing surface character, localizing on the sides perpendicular to the direction of the magnetic field–see, e.g. Figures 4, 6 ([110] and [100]), and in the Supporting Information Figure S2 (modes no. 7 and 8) and Figure S3 (modes no. 1 and 5). This may be the result of an additional effect arising from the strong influence of crystallography and the shape anisotropy on the localization of resonant modes in different regions and probably on the chirality of the system. However, the analysis of these effects, although interesting, is beyond the scope of this work, since it is necessary to perform micromagnetic simulations with periodic boundary conditions and to conduct experiments of a different type, where the sample containing the single-domain gyroidal structure could be selectively analyzed on a much smaller scale.

In addition, the distinct mode spectrum observed in our research has direct implications for radio frequency filtering applications. The presence of a singular bulk mode in both BBFMR experiments (as an effective response from a multidomain structure) or simulations (a strong resonance in a single domain) enables the design of selective filter systems. They could be tailored to isolate specific FMR frequencies depending on factors such as crystallographic orientation relative to the incident signal and filling factor. In addition, the three-dimensional magnonic structures are expected to exhibit superior absorption properties compared to their two-dimensional counterparts, as supported by previous studies.^44^

## 6 Conclusions

In the investigation, a comprehensive ferromagnetic resonance analysis was conducted on three-dimensional gyroidal Ni nanostructures, delving into the complex interplay between magnetic properties and structural geometry. Utilizing FEM micromagnetic simulations in the time domain to determine the static magnetization structures^55^ and in the frequency domain to investigate their oscillatory, magnonic properties,^57^ the study embarked on a multifaceted exploration aimed not merely at interpreting experimental results but also at unraveling the intricate magnetization distribution within the gyroid nanostructure.

A major result that emerges from our simulations is the importance of the crystallographic orientation of the gyroid, relative to the magnetic field’s direction, on a frequency and spatial distribution of the collective magnetization oscillations. Complementing these simulations, experimental measurements were executed using BBFMR, where a multidomain gyroid sample was positioned on a CPW line, enabling analysis of differences and relationships between the solid, uniform Ni layer and the gyroid-structured portion of the sample. Although the measurements were not able to study individual crystallographic domains of the gyroid and thus confirm all simulation predictions, based on our findings, gyroid films can be conceptualized as homogeneous materials, i.e., magnonic metamaterials, where the effective saturation magnetization is reduced by the gyroid filling factor and the FMR signal line width encapsulates more than the inherent damping properties of the material; it is also intricately linked to the particular geometry and crystallographic orientation of the structure.

Collectively, these discoveries show a new frontier in the realm of 3D magnonics, positing the gyroid structure as a potential cornerstone in the field. The results not only augment our theoretical and experimental grasp of the magnetization dynamics in complex nanostructures but also open up promising avenues for practical applications, imbuing the gyroid configuration with the potential to become an elemental building block in emerging magnetic technologies. We propose a gyroidal structure with a tunable filling factor as a magnonic crystal and as the basis for novel 3D radio frequency filters. The study’s interdisciplinary approach, bridging numerical simulations with empirical investigation, marks a significant stride toward the comprehensive understanding and manipulation of magnetic resonance in three-dimensional architectures.

## Data Availability

The data underlying this study are openly available in Zenodo at https://doi.org/10.5281/zenodo.11004007.

## Supporting Information Available

BBFMR gyroid structure analysis under in-plane field rotation in 40° increments over 180° relative to the CPW; micromagnetic results of 4 × 4 × 4 gyroid spectra at 100 mT: detailed focus on satellite peaks; micromagnetic results of 4 × 4 × 4 gyroid spectra at 300 mT: detailed focus on satellite peaks; and FWHM vs frequency: comparison of BBFMR signals of gyroids and uniform Ni, under sample rotations on CPW, revealing the variation in the resulting effective parameters of damping and inhomogeneous contributions to the line width (PDF)

## References

[ref1] Pirro P.; Vasyuchka V. I.; Serga A. A.; Hillebrands B. Advances in coherent magnonics. Nat. Rev. Mater. 2021, 6, 1114–1135. 10.1038/s41578-021-00332-w.

[ref2] Serga A. A.; Chumak A. V.; Hillebrands B. YIG magnonics. J. Phys. D: Appl. Phys. 2010, 43, 264002 10.1088/0022-3727/43/26/264002.

[ref3] Yan P.; Bauer G. E. Magnon mediated domain wall heat conductance in ferromagnetic wires. IEEE Trans. Magn. 2013, 49, 3109–3112. 10.1109/TMAG.2013.2249577.

[ref4] Garcia-Sanchez F.; Borys P.; Soucaille R.; Adam J. P.; Stamps R. L.; Kim J. V. Narrow Magnonic Waveguides Based on Domain Walls. Phys. Rev. Lett. 2015, 114, 247206 10.1103/PhysRevLett.114.247206. 26197006

[ref5] Wagner K.; Kákay A.; Schultheiss K.; Henschke A.; Sebastian T.; Schultheiss H. Magnetic domain walls as reconfigurable spin-wave nanochannels. Nat. Nanotechnol. 2016, 11, 432–436. 10.1038/nnano.2015.339. 26828849

[ref6] Chumak A. V.; Vasyuchka V. I.; Serga A. A.; Hillebrands B. Magnon spintronics. Nat. Phys. 2015, 11, 453–461. 10.1038/nphys3347.

[ref7] Kruglyak V. V.; Demokritov S. O.; Grundler D. Magnonics. J. Phys. D: Appl. Phys. 2010, 43, 264001 10.1088/0022-3727/43/26/264001.

[ref8] Barman A.; Gubbiotti G.; Ladak S.; Adeyeye A. O.; Krawczyk M.; Gräfe J.; Adelmann C.; Cotofana S.; Naeemi A.; Vasyuchka V. I.; et al. The 2021 Magnonics Roadmap. J. Phys.: Condens. Matter 2021, 33, 413001 10.1088/1361-648x/abec1a. 33662946

[ref9] Chumak A. V.; Kabos P.; Wu M.; Abert C.; Adelmann C.; Adeyeye A. O.; Akerman J.; Aliev F. G.; Anane A.; Awad A.; et al. Advances in Magnetics Roadmap on Spin-Wave Computing. IEEE Trans. Magn. 2022, 58, 1–72. 10.1109/tmag.2022.3149664.

[ref10] Gubbiotti G. In Three-Dimensional Magnonics, 1st ed.; Gubbiotti G., Ed.; Jenny Stanford Publishing: New York, 2019.

[ref11] Cheenikundil R.; d’Aquino M.; Hertel R. Defect-sensitive High-frequency Modes in a Three-Dimensional Artificial Magnetic Crystal. arXiv 2023, arXiv.2312.08415 10.48550/arXiv.2312.08415.

[ref12] Krawczyk M.; Grundler D. Review and prospects of magnonic crystals and devices with reprogrammable band structure. J. Phys.: Condens. Matter 2014, 26, 123202 10.1088/0953-8984/26/12/123202. 24599025

[ref13] Makarov D.; Volkov O. M.; Kákay A.; Pylypovskyi O. V.; Budinská B.; Dobrovolskiy O. V. New Dimension in Magnetism and Superconductivity: 3D and Curvilinear Nanoarchitectures. Adv. Mater. 2022, 34, 2101758 10.1002/adma.202101758. PMC11469131 34705309

[ref14] Cheenikundil R.; Bauer J.; Goharyan M.; d’Aquino M.; Hertel R. High-frequency modes in a magnetic buckyball nanoarchitecture. APL Mater. 2022, 10, 81106 10.1063/5.0097695.

[ref15] Cheenikundil R.; d’Aquino M.; Hertel R. Magnetization dynamics in a three-dimensional interconnected nanowire array. arXiv 2023, arXiv.2306.00174 10.48550/arXiv.2306.00174.

[ref16] Llandro J.; Love D. M.; Kovács A.; Caron J.; Vyas K. N.; Kákay A.; Salikhov R.; Lenz K.; Fassbender J.; Scherer M. R. J.; et al. Visualizing magnetic structure in 3d nanoscale ni-fe gyroid networks. Nano Lett. 2020, 20, 3642–3650. 10.1021/acs.nanolett.0c00578. 32250635

[ref17] Fernández-Pacheco A.; Streubel R.; Fruchart O.; Hertel R.; Fischer P.; Cowburn R. P. Three-dimensional nanomagnetism. Nat. Commun. 2017, 8, 15756 10.1038/ncomms15756. 28598416 PMC5494189

[ref18] Donnelly C.; Hierro-Rodríguez A.; Abert C.; Witte K.; Skoric L.; Sanz-Hernández D.; Finizio S.; Meng F.; McVitie S.; Raabe J.; Suess D.; Cowburn R.; Fernández-Pacheco A. Complex free-space magnetic field textures induced by three-dimensional magnetic nanostructures. Nat. Nanotechnol. 2022, 17, 136–142. 10.1038/s41565-021-01027-7. 34931031 PMC8850196

[ref19] van den Berg A.; Caruel M.; Hunt M.; Ladak S. Combining two-photon lithography with laser ablation of sacrificial layers: A route to isolated 3D magnetic nanostructures. Nano Res. 2023, 16, 1441–1447. 10.1007/s12274-022-4649-z.

[ref20] Schoen A. H. Infinite periodic minimal surfaces without self-intersections; National Aeronautics and Space Administration, 1970.

[ref21] Lambert C. A.; Radzilowski L. H.; Thomas E. L. Triply periodic level surfaces as models for cubic tricontinuous block copolymer morphologies. Philos. Trans. R. Soc., A 1996, 354, 2009–2023. 10.1098/rsta.1996.0089.

[ref22] Turner M. D.; Saba M.; Zhang Q.; Cumming B. P.; Schröder-Turk G. E.; Gu M. Miniature chiral beamsplitter based on gyroid photonic crystals. Nat. Photonics 2013, 7, 801–805. 10.1038/nphoton.2013.233.

[ref23] Vignolini S.; Yufa N. A.; Cunha P. S.; Guldin S.; Rushkin I.; Stefik M.; Hur K.; Wiesner U.; Baumberg J. J.; Steiner U. A 3D optical metamaterial made by self-assembly. Adv. Mater. 2012, 24, OP23–OP27. 10.1002/adma.201103610. 22021112

[ref24] Dolan J. A.; Wilts B. D.; Vignolini S.; Baumberg J. J.; Steiner U.; Wilkinson T. D. Optical Properties of Gyroid Structured Materials: From Photonic Crystals to Metamaterials. Adv. Opt. Mater. 2015, 3, 12–32. 10.1002/adom.201400333.

[ref25] Michielsen K.; Stavenga D. G. Gyroid cuticular structures in butterfly wing scales: Biological photonic crystals. J. R. Soc., Interface 2008, 5, 85–94. 10.1098/rsif.2007.1065. 17567555 PMC2709202

[ref26] Saranathan V.; Osuji C. O.; Mochrie S. G.; Noh H.; Narayanan S.; Sandy A.; Dufresne E. R.; Prum R. O. Structure, function, and self-assembly of single network gyroid (I4 132) photonic crystals in butterfly wing scales. Proc. Natl. Acad. Sci. U.S.A. 2010, 107, 11676–11681. 10.1073/pnas.0909616107. 20547870 PMC2900708

[ref27] Schröder-Turk G.; Wickham S.; Averdunk H.; Brink F.; Fitz Gerald J. D.; Poladian L.; Large M. C.; Hyde S. T. The chiral structure of porous chitin within the wing-scales of Callophrys rubi. J. Struct. Biol. 2011, 174, 290–295. 10.1016/j.jsb.2011.01.004. 21272646

[ref28] Yan C.; Hao L.; Hussein A.; Raymont D. Evaluations of cellular lattice structures manufactured using selective laser melting. Int. J. Mach. Tool Manufact. 2012, 62, 32–38. 10.1016/j.ijmachtools.2012.06.002.

[ref29] Yánez A.; Herrera A.; Martel O.; Monopoli D.; Afonso H. Compressive behaviour of gyroid lattice structures for human cancellous bone implant applications. Mater. Sci. Eng., C 2016, 68, 445–448. 10.1016/j.msec.2016.06.016. 27524040

[ref30] Armatas G. S.; Kanatzidis M. G. Mesostructured germanium with cubic pore symmetry. Nature 2006, 441, 1122–1125. 10.1038/nature04833. 16810250

[ref31] Hajduk D. A.; Harper P. E.; Gruner S. M.; Honeker C. C.; Kim G.; Thomas E. L.; Kim G. The Gyroid: A New Equilibrium Morphology in Weakly Segregated Diblock Copolymers. Macromolecules 1994, 27, 4063–4075. 10.1021/ma00093a006.

[ref32] Kim J. K.; Yang S. Y.; Lee Y.; Kim Y. Functional nanomaterials based on block copolymer self-assembly. Prog. Polym. Sci. 2010, 35, 1325–1349. 10.1016/j.progpolymsci.2010.06.002.

[ref33] Bai W.; Hannon A. F.; Gotrik K. W.; Choi H. K.; Aissou K.; Liontos G.; Ntetsikas K.; Alexander-Katz A.; Avgeropoulos A.; Ross C. A. Thin film morphologies of bulk-gyroid polystyrene-block-polydimethylsiloxane under solvent vapor annealing. Macromolecules 2014, 47, 6000–6008. 10.1021/ma501293n.

[ref34] Hsueh H. Y.; Yao C. T.; Ho R. M. Well-ordered nanohybrids and nanoporous materials from gyroid block copolymer templates. Chem. Soc. Rev. 2015, 44, 1974–2018. 10.1039/C4CS00424H. 25622806

[ref35] Lich L. V.; Hue D. T. H.; Giang D. T. H.; Duc N. H.; Shimada T.; Kitamura T.; Dinh V. H. Formation and switching of chiral magnetic field textures in three-dimensional gyroid nanostructures. Acta Mater. 2023, 249, 118802 10.1016/j.actamat.2023.118802.

[ref36] Hertel R. Curvature-induced magnetochirality. SPIN 2013, 03, 1340009.

[ref37] Gaididei Y.; Kravchuk V. P.; Sheka D. D. Curvature Effects in Thin Magnetic Shells. Phys. Rev. Lett. 2014, 112, 257203 10.1103/PhysRevLett.112.257203. 25014827

[ref38] Streubel R.; Fischer P.; Kronast F.; Kravchuk V. P.; Sheka D. D.; Gaididei Y.; Schmidt O. G.; Makarov D. Magnetism in curved geometries. J. Phys. D: Appl. Phys. 2016, 49, 363001 10.1088/0022-3727/49/36/363001.

[ref39] Sander D.; Valenzuela S. O.; Makarov D.; Marrows C. H.; Fullerton E. E.; Fischer P.; McCord J.; Vavassori P.; Mangin S.; Pirro P.; et al. The 2017 Magnetism Roadmap. J. Phys. D: Appl. Phys. 2017, 50, 363001 10.1088/1361-6463/aa81a1.

[ref40] Sheka D. D. A perspective on curvilinear magnetism. Appl. Phys. Lett. 2021, 118, 230502 10.1063/5.0048891.

[ref41] Shindou R.; Matsumoto R.; Murakami S.; Ohe J. I. Topological chiral magnonic edge mode in a magnonic crystal. Phys. Rev. B: Condens. Matter Mater. Phys. 2013, 87, 174427 10.1103/PhysRevB.87.174427.

[ref42] McClarty P. A. Topological Magnons: A Review. Annu. Rev. Condens. Matter Phys. 2022, 13, 171–190. 10.1146/annurev-conmatphys-031620-104715.

[ref43] May A.; Saccone M.; van den Berg A.; Askey J.; Hunt M.; Ladak S. Magnetic charge propagation upon a 3D artificial spin-ice. Nat. Commun. 2021, 12, 3217–3310. 10.1038/s41467-021-23480-7. 34050163 PMC8163774

[ref44] Guo H.; Deenen A. J. M.; Xu M.; Hamdi M.; Grundler D. Realization and Control of Bulk and Surface Modes in 3D Nanomagnonic Networks by Additive Manufacturing of Ferromagnets. Adv. Mater. 2023, 35, 2303292 10.1002/adma.202303292. 37450937

[ref45] Krawczyk M.; Puszkarski H. Magnonic crystal theory of the spin-wave frequency gap in low-doped manganites. J. Appl. Phys. 2006, 100, 073905 10.1063/1.2356082.

[ref46] Krawczyk M.; Puszkarski H. Plane-wave theory of three-dimensional magnonic crystals. Phys. Rev. B: Condens. Matter Mater. Phys. 2008, 77, 054437 10.1103/PhysRevB.77.054437.

[ref47] Volkov O. M.; Rößler U. K.; Fassbender J.; Makarov D. Concept of artificial magnetoelectric materials via geometrically controlling curvilinear helimagnets. J. Phys. D: Appl. Phys. 2019, 52, 345001 10.1088/1361-6463/ab2368.

[ref48] Hopfield J. J. Neural networks and physical systems with emergent collective computational abilities. Proc. Natl. Acad. Sci. U.S.A. 1982, 79, 2554–2558. 10.1073/pnas.79.8.2554. 6953413 PMC346238

[ref49] Karcher H. The triply periodic minimal surfaces of Alan Schoen and their constant mean curvature companions. Manuscripta Math. 1989, 64, 291–357. 10.1007/BF01165824.

[ref50] Große-Brauckmann K.; Wohlgemuth M. The gyroid is embedded and has constant mean curvature companions. Calc. Var. Partial Differ. Equ. 1996, 4, 499–523. 10.1007/s005260050052.

[ref51] Große-Brauckmann K. Gyroids of constant mean curvature. Exp. Math. 1997, 6, 33–50. 10.1080/10586458.1997.10504349.

[ref52] Sunada T. Crystals That Nature Might Miss Creating. Not. AMS 2008, 55, 208–215.

[ref53] Hyde S. T.; O’Keeffe M.; Proserpio D. M. A short history of an elusive yet ubiquitous structure in chemistry, materials, and mathematics. Angew. Chem., Int. Ed. 2008, 47, 7996–8000. 10.1002/anie.200801519. 18767088

[ref54] Coey J. M. Magnetism and Magnetic Materials; Cambridge University Press, 2010; pp 1–617.

[ref55] Hertel R. tetmag. 2023, https://github.com/R-Hertel/tetmag.

[ref56] Hertel R.; Christophersen S.; Börm S. Large-scale magnetostatic field calculation in finite element micromagnetics with H 2 -matrices. J. Magn. Magn. Mater. 2019, 477, 118–123. 10.1016/j.jmmm.2018.12.103.

[ref57] d’Aquino M.; Hertel R. Micromagnetic frequency-domain simulation methods for magnonic systems. J. Appl. Phys. 2023, 133, 033902 10.1063/5.0131922.

[ref58] d’Aquino M.; Serpico C.; Miano G.; Forestiere C. A novel formulation for the numerical computation of magnetization modes in complex micromagnetic systems. J. Comput. Phys. 2009, 228, 6130–6149. 10.1016/j.jcp.2009.05.026.

[ref59] Heinrich B. In Ultrathin Magnetic Structures II Heinrich B., Bland J., Eds.; Springer: Berlin, Heidelberg, 1994; pp 195–296.

[ref60] Montoya E.; McKinnon T.; Zamani A.; Girt E.; Heinrich B. Broadband ferromagnetic resonance system and methods for ultrathin magnetic films. J. Magn. Magn. Mater. 2014, 356, 12–20. 10.1016/j.jmmm.2013.12.032.

[ref61] Dubowik J.; Głowiński H. Broad-Band Ferromagnetic Resonance in Thin Magnetic Films and Nanostructures. Current Topics in Biophysics 2010, 33, 43–45.

[ref62] Mikhaylovskiy R. V.; Hendry E.; Kruglyak V. V. Negative permeability due to exchange spin-wave resonances in thin magnetic films with surface pinning. Phys. Rev. B: Condens. Matter Mater. Phys. 2010, 82, 195446 10.1103/PhysRevB.82.195446.

[ref63] Mruczkiewicz M.; Krawczyk M.; Mikhaylovskiy R. V.; Kruglyak V. V. Towards high-frequency negative permeability using magnonic crystals in metamaterial design. Phys. Rev. B: Condens. Matter Mater. Phys. 2012, 86, 024425 10.1103/PhysRevB.86.024425.

[ref64] Kruglyak V.; In Metamaterial; Jiang X.-Y., Ed.; IntechOpen: Rijeka, 2012; Chapter 14.

[ref65] Zhuo F.; Li H.; Cheng Z.; Manchon A. Magnonic Metamaterials for Spin-Wave Control with Inhomogeneous Dzyaloshinskii-Moriya Interactions. Nanomaterials 2022, 12, 1159 10.3390/nano12071159. 35407277 PMC9000796

[ref66] Haldar A.; Adeyeye A. O. Reconfigurable and self-biased magnonic metamaterials. J. Appl. Phys. 2020, 128, 240902 10.1063/5.0033254.

[ref67] Beaujour J.-M.; Ravelosona D.; Tudosa I.; Fullerton E. E.; Kent A. D. Ferromagnetic resonance linewidth in ultrathin films with perpendicular magnetic anisotropy. Phys. Rev. B: Condens. Matter Mater. Phys. 2009, 80, 180415 10.1103/PhysRevB.80.180415.

[ref68] Walowski J.; Kaufmann M. D.; Lenk B.; Hamann C.; McCord J.; Münzenberg M. Intrinsic and non-local Gilbert damping in polycrystalline nickel studied by Ti: sapphire laser fs spectroscopy. J. Phys. D: Appl. Phys. 2008, 41, 164016 10.1088/0022-3727/41/16/164016.

[ref69] Abdelrahman D.; Iseli R.; Musya M.; Jinnai B.; Fukami S.; Yuasa T.; Sai H.; Wiesner U. B.; Saba M.; Wilts B. D.; Steiner U.; Llandro J.; Gunkel I. Directed Self-Assembly of Diamond Networks in Triblock Terpolymer Films on Patterned Substrates. ACS Appl. Mater. Interfaces 2023, 15, 57981–57991. 10.1021/acsami.3c10619. 37989271 PMC10739600

[ref70] Gurevich A.; Melkov G. Magnetization Oscillations and Waves; Taylor & Francis, 1996.

