# Supplementary material for: Collective Spin-Wave Dynamics in Gyroid Ferromagnetic Nanostructures
Source: ACS Appl Mater Interfaces. 2024 Apr 22;16(17):22177–88. doi: 10.1021/acsami.4c02366 (PMC11071044; doi:10.1021/acsami.4c02366)
Supplement: Supplementary file 1 — am4c02366_si_001.pdf [file am4c02366_si_001.pdf]

Supporting Information: Collective Spin-Wave Dynamics in Gyroid Ferromagnetic Nanostructures

Mateusz Gołębiewski,[*,†] Riccardo Hertel,[‡] Massimiliano d'Aquino,[¶] Vitaliy Vasyuchka,[§] Mathias Weiler,[§] Philipp Pirro,[§] Maciej Krawczyk,[†] Shunsuke Fukami,[‖,⊥,#,@,△] Hideo Ohno,[‖,⊥,#,@] and Justin Llandro[‖,⊥]

†*Institute of Spintronics and Quantum Information, Faculty of Physics, Adam Mickiewicz University, Uniwersytetu Poznańskiego 2, 61-614 Poznań, Poland*

‡*Université de Strasbourg, CNRS, Institut de Physique et Chimie des Matériaux de Strasbourg, F-67000 Strasbourg, France*

¶*Department of Electrical Engineering and ICT, University of Naples Federico II, 80125 Naples, Italy*

§*Fachbereich Physik und Landesforschungszentrum OPTIMAS, Rheinland-Pfälzische Technische Universität Kaiserslautern-Landau, Erwin-Schrödinger-Straße 56, 67663 Kaiserslautern, Germany*

‖*Research Institute of Electrical Communication (RIEC), Tohoku University, 2-1-1 Katahira, Aoba-ku, 980-8577 Sendai-shi Miyagi, Japan*

⊥*Center for Science and Innovation in Spintronics (CSIS), Tohoku University, 980-8577 Sendai, Japan*

#*Center for Innovative Integrated Electronic Systems (CIES), Tohoku University, 468-1 Aramaki Aza Aoba, Aoba-ku, 980-0845 Sendai, Japan*

@*WPI Advanced Institute for Materials Research, Tohoku University, 2-1-1 Katahira, Aoba-ku, 980-8577 Sendai, Japan*

△*Inamori Research Institute for Science, 600-8411 Kyoto, Japan*

E-mail: mateusz.golebiewski@amu.edu.pl

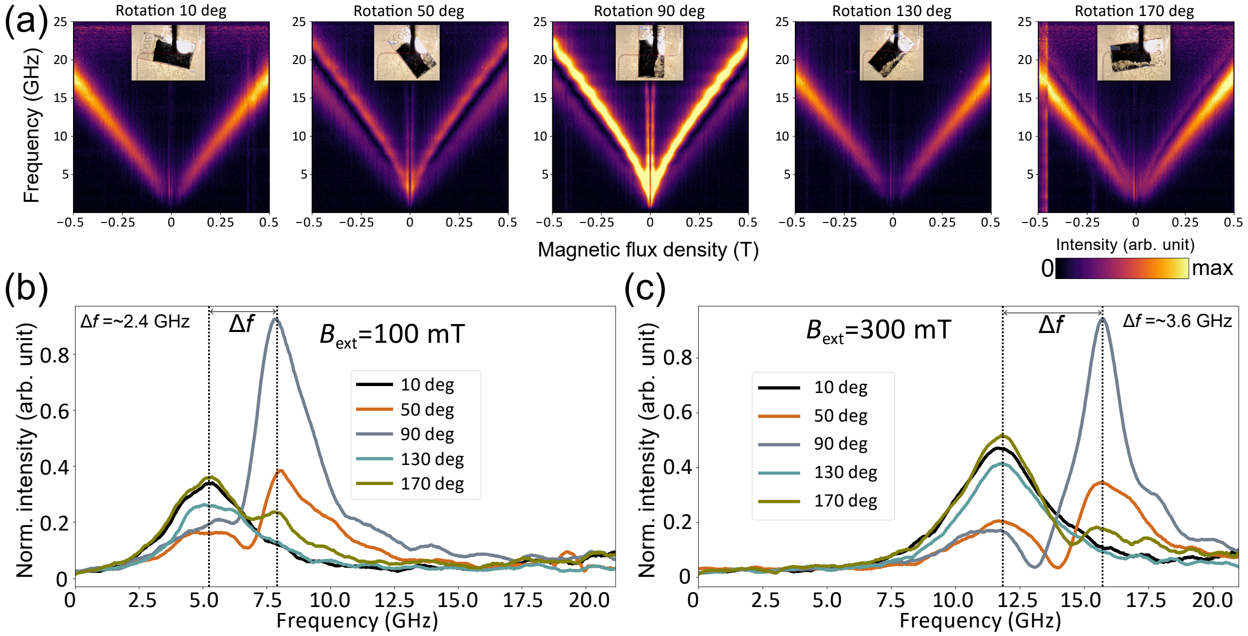

Figure S1: BBFMR measurement of the gyroid structure. The sample was rotated 180 deg relative to the CPW in 40 deg steps. For each configuration, a separate measurement of the energy absorption from the microwave field applied perpendicular to the static external magnetic field was performed as shown in (a). Plots of the FMR intensity as a function of frequency (for $B_{\text{ext}} = 100$ mT and $B_{\text{ext}} = 300$ mT) for selected angles are presented in (b) and (c), respectively. They show a clear and cyclic transition of spectral weight from the lower frequency branch from gyroidal sample's zone (high intensity at 10 deg) to the higher one from uniform Ni (high intensity at 90 deg). The separation between the bands (Δf) are measured to be 2.64 GHz and 3.89 GHz, respectively, as the field increases. Unlike amplitude, Δf is independent of rotation angle. The assumed values of the angles are conventional and do not refer to the crystallographic axes of the gyroid.

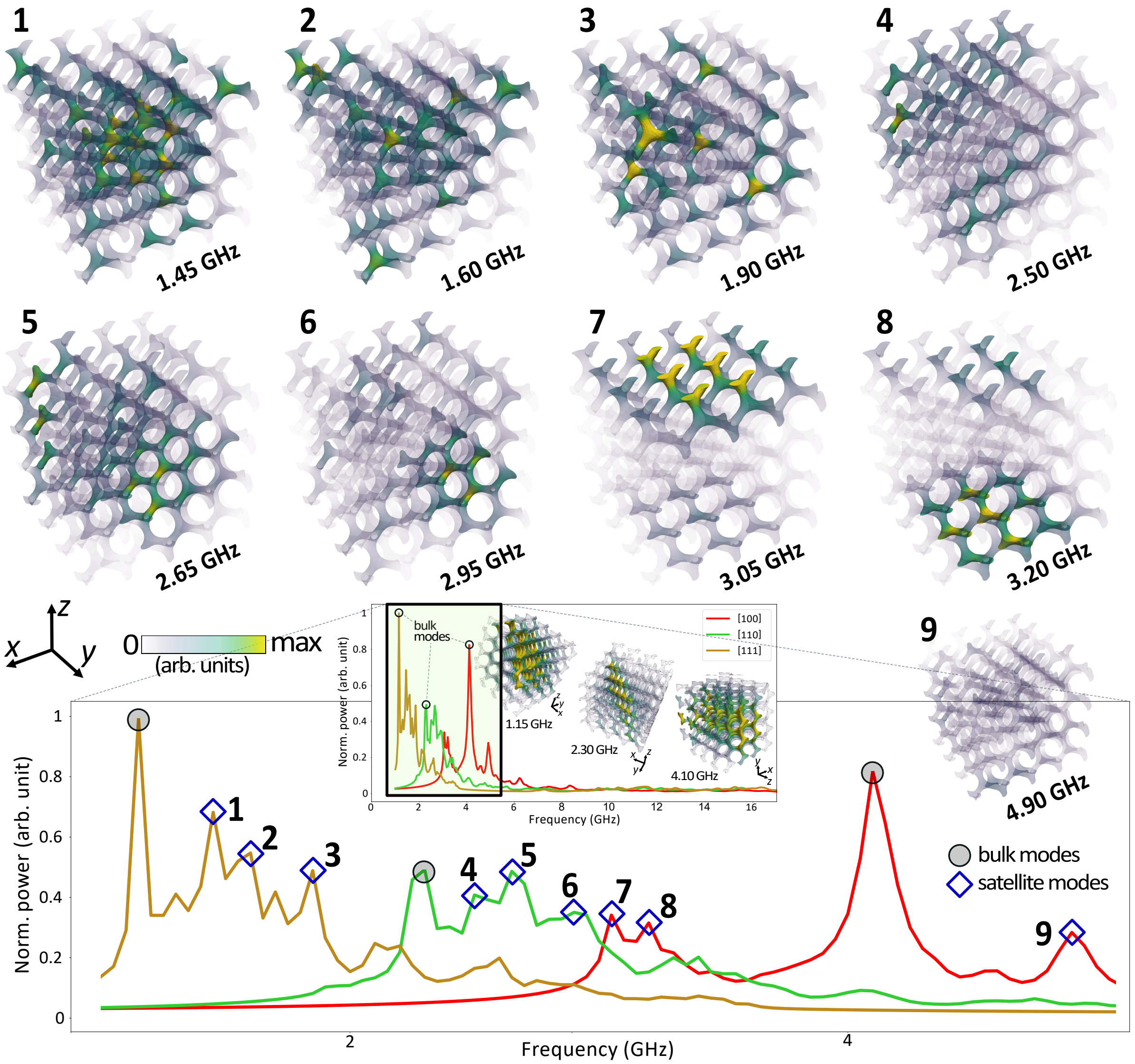

Figure S2: Micromagnetic simulation-derived resonance frequency spectra for $4 \times 4 \times 4$ gyroid constructs in which the applied external magnetic field has a strength of 100 mT. The spectrum used in the main part of the work has been enlarged, focusing on each individual satellite peaks. Different color coding in the plot indicates the crystallographic direction in which the field is applied, with the specific points marked on the plot indicating all significant satellite/edge ferromagnetic modes (diamonds) and volumetric modes (gray circles). Each satellite mode is numbered according to the visualization of the respective module with its frequency values above. The color scheme used here corresponds directly to the imaginary part of the magnetic susceptibility.

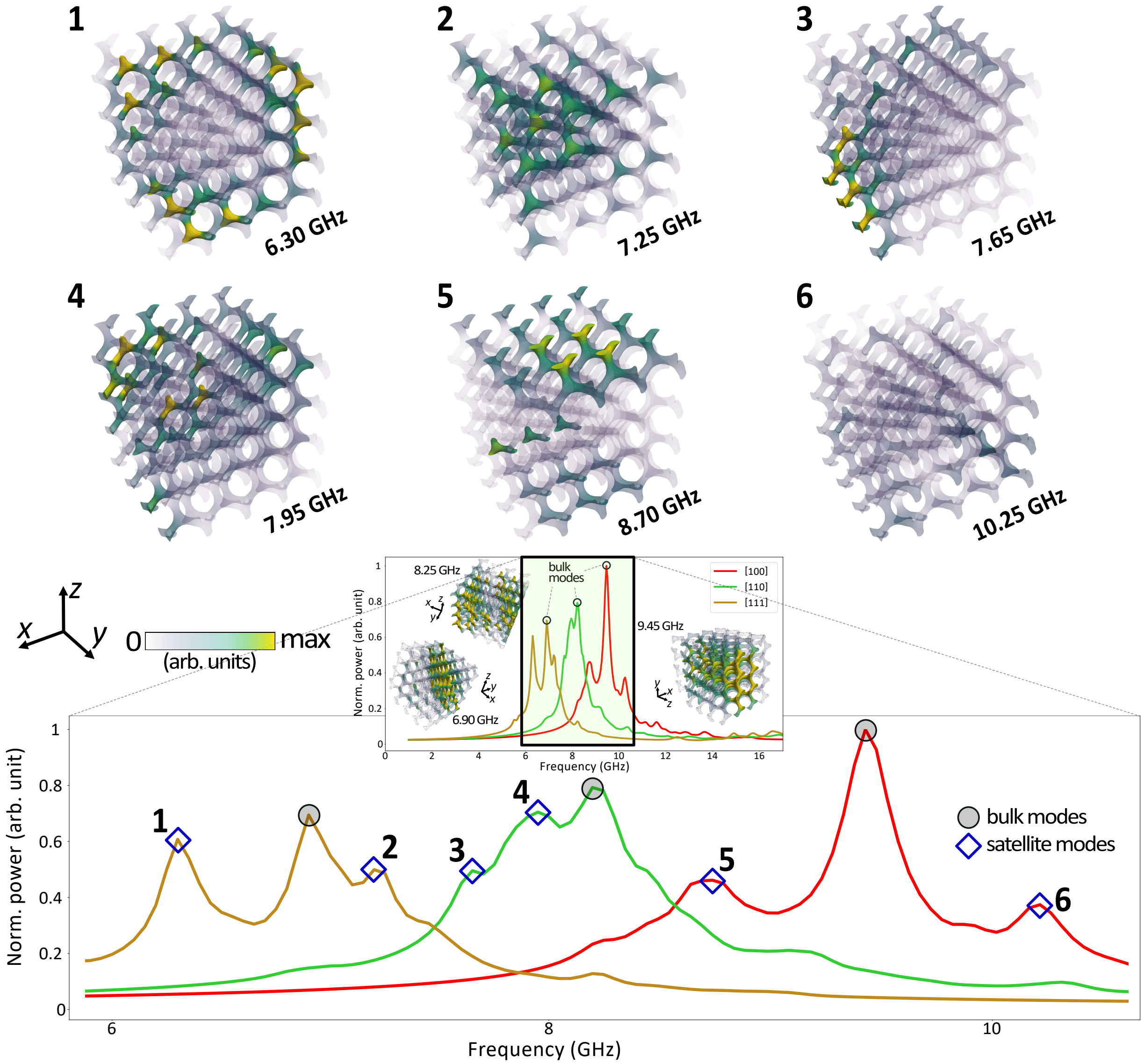

Figure S3: Micromagnetic simulation-derived resonance frequency spectra for $4 \times 4 \times 4$ gyroid constructs in which the applied external magnetic field has a strength of 300 mT. The spectrum used in the main part of the work has been enlarged, focusing on each individual satellite peaks. Different color coding in the plot indicates the crystallographic direction in which the field is applied, with the specific points marked on the plot indicating all significant satellite/edge ferromagnetic modes (diamonds) and volumetric modes (gray circles). Each satellite mode is numbered according to the visualization of the respective module with its frequency values above. The color scheme used here corresponds directly to the imaginary part of the magnetic susceptibility.

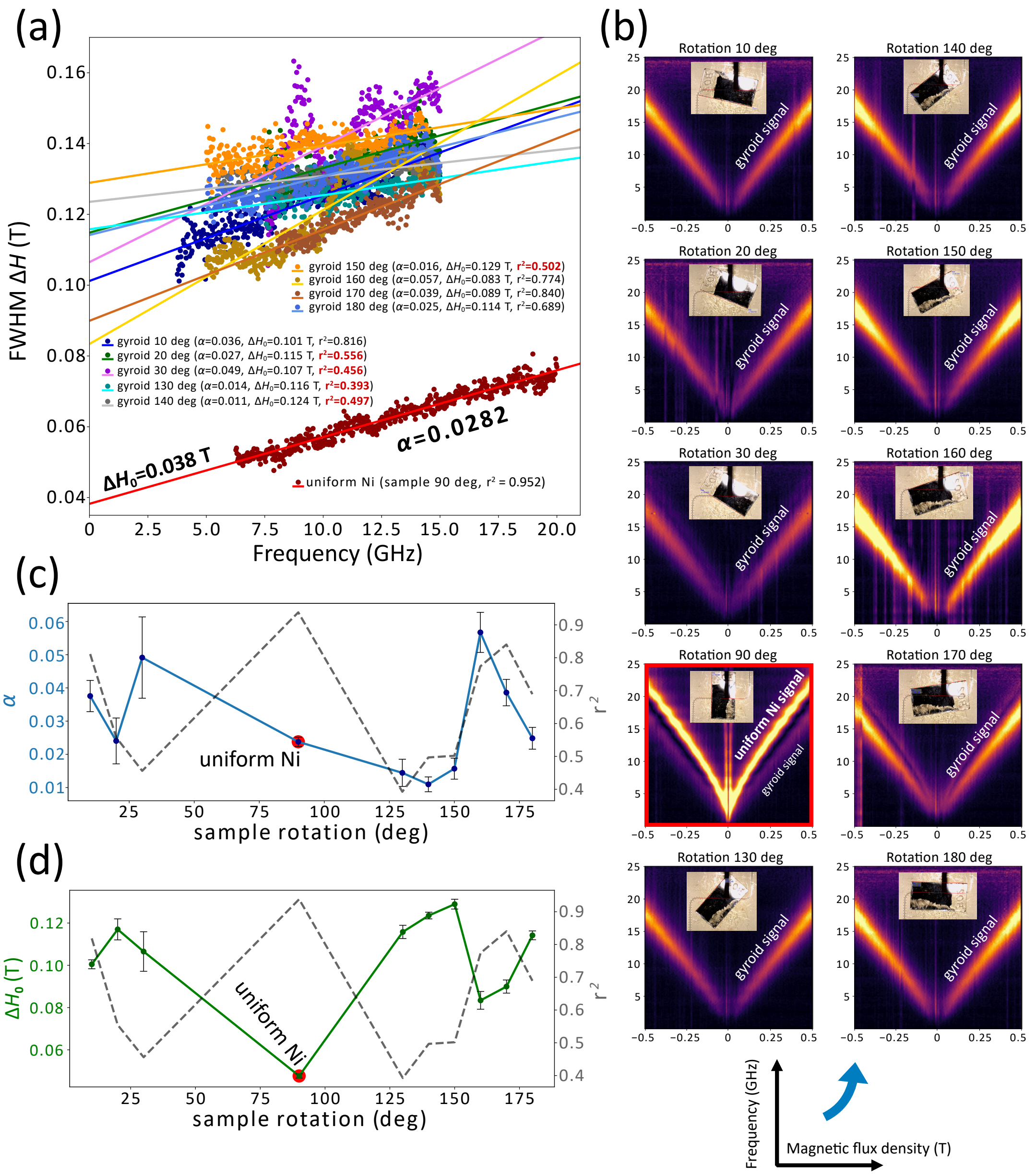

Figure S4: Plot (a) shows the FWHM values as a function of frequency, comparing BBFMR signals from gyroidal Ni and uniform Ni (represented by dark red dots). Several signals from gyroids were obtained by rotating the sample on the CPW, as illustrated in Figure S1, effectively obtaining a signal from various domains with different crystallography. Based on the experimental results and using Equation 3 from the main manuscript, linear regressions were performed on each set of data. The estimated values of the determination coefficient r^2, inhomogeneous contributions to the linewidth ΔH_0 (measured from the abscissa of the lines), and the damping values α (derived from the slope of the lines) were estimated. (b) illustrates BBFMR measurements of dynamic magnetization intensities as functions of static magnetic flux density and frequency for selected sample configurations. The gyroid signal dominates all of them except for the 90-deg rotation, which is indicated by the red box. The changes in α and ΔH_0 as a function of sample rotation relative to the CPW with corresponding statistical error-bars are presented in (c) and (d). The curves with the values of the coefficient of determination r^2 for the measured configurations are additionally plotted on the graphs, showing their strongly nonlinear/irregular nature for some of the measurements [the lowest quality r^2 values are highlighted in red in (a)]. In the main manuscript, configurations with reliable linear regression fits were used, marked here with angles of 10 deg (for gyroid signal analysis) and 90 deg (for homogeneous Ni signal analysis).
